# Treating Multiple Myeloma in the Context of the Bone Marrow Microenvironment

**DOI:** 10.3390/curroncol29110705

**Published:** 2022-11-21

**Authors:** Matthew Ho, Alexander Xiao, Dongni Yi, Saurabh Zanwar, Giada Bianchi

**Affiliations:** 1 Department of Internal Medicine, Mayo Clinic, Rochester, MN 55902, USA; 2 Division of Hematology, Department of Internal Medicine, Mayo Clinic, Rochester, MN 55902, USA; 3 Division of Hematology, Brigham and Women’s Hospital, Harvard Medical School, Boston, MA 02120, USA

**Keywords:** multiple myeloma, bone marrow microenvironment, immunotherapy, chimeric antigen receptor-T cells, bispecific T-cell engager

## Abstract

The treatment landscape of multiple myeloma (MM) has evolved considerably with the FDA-approval of at least 15 drugs over the past two decades. Together with the use of autologous stem cell transplantation, these novel therapies have resulted in significant survival benefit for patients with MM. In particular, our improved understanding of the BM and immune microenvironment has led to the development of highly effective immunotherapies that have demonstrated unprecedented response rates even in the multiple refractory disease setting. However, MM remains challenging to treat especially in a high-risk setting. A key mediator of therapeutic resistance in MM is the bone marrow (BM) microenvironment; a deeper understanding is necessary to facilitate the development of therapies that target MM in the context of the BM milieu to elicit deeper and more durable responses with the ultimate goal of long-term control or a cure of MM. In this review, we discuss our current understanding of the role the BM microenvironment plays in MM pathogenesis, with a focus on its immunosuppressive nature. We also review FDA-approved immunotherapies currently in clinical use and highlight promising immunotherapeutic approaches on the horizon.

## 1. Introduction

**Figure 1:**
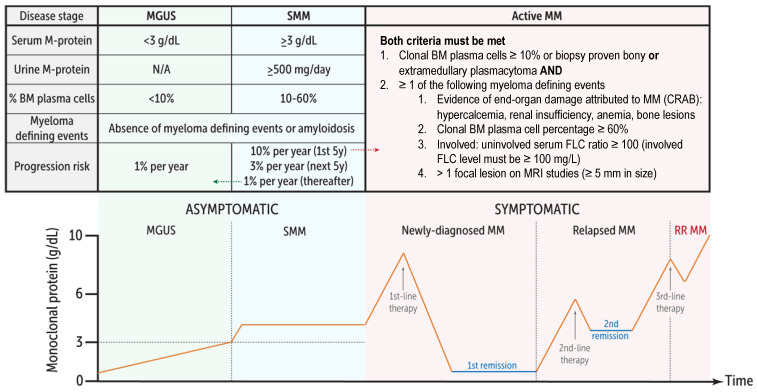
Multiple myeloma and asymptomatic precursor states. MM is preceded in virtually all cases by the asymptomatic precursor states monoclonal gammopathy of undetermined significance (MGUS) and smoldering multiple myeloma (SMM). The international myeloma working group (IMWG) defines asymptomatic MM as having ≥1 myeloma defining events with either clonal BM plasma cells ≥10% or biopsy proven plasmacytoma.

**Figure 2:**
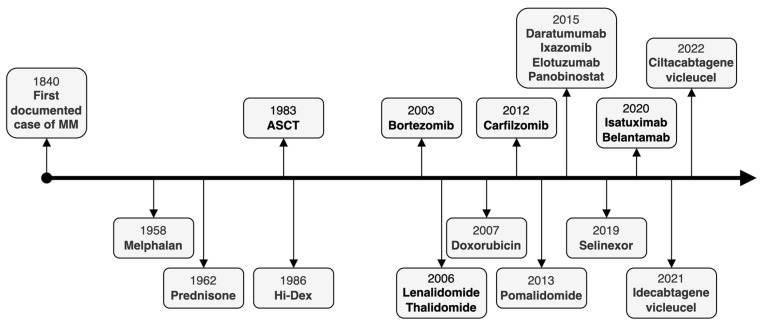
Timeline of treatment advancements in multiple myeloma.

Multiple myeloma (MM) is the second most common hematologic malignancy in high-income countries [1]. MM is preceded in virtually all cases by the asymptomatic precursor states monoclonal gammopathy of undetermined significance (MGUS) and smoldering multiple myeloma (SMM) (Figure 1), as shown by population-based studies published in 2009 [2,3]. Survival in MM has improved significantly over the past decades with the use of autologous stem cell transplantation (ASCT), as well as novel agents that treat MM in the context of the BM microenvironment (Figure 2) [4]. However, MM is still mostly incurable outside of allogeneic stem cell transplant which, despite significant treatment-related toxicity, remains the only treatment that can enable long-term progression-free survival (PFS) or potentially even cure carefully selected patients with high-risk MM [5]. Similar to how long-lived plasma cells are critically dependent on their ability to traffic to pro-survival niches within the BM and other lymphoid organs [6], it has become increasingly clear that MM cells rely on the BM microenvironment for survival, proliferation, and drug resistance [7,8]. This reliance on the BM microenvironment occurs early on during the MGUS phase as suggested by studies showing progressive growth in MGUS cells xenotransplanted in humanized mice that express several human genes essential for the growth of human cells [9]. Additionally, a recent whole-genome analysis of paired samples from a small number of non-high-risk SMM cases that progressed to MM revealed that, in some cases, the SMM clone may be genomically indistinguishable from MM [10]. This further suggests that the BM microenvironment or tumor-extrinsic signals may play a dominant role in influencing MM growth and progression. This MM-permissive BM microenvironment includes noncellular components (e.g., extracellular matrix proteins, growth factors, cytokines, extracellular vesicles), which are produced or influenced by the cellular compartments comprising BM mesenchymal stromal cells, immune cells, osteoblasts/osteoclasts, and BM endothelial cells [8].

Figure 2 summarizes the timeline of FDA approvals for therapies for multiple myeloma.

## 2. The Role of the Tumor Microenvironment in the Initiation of the Myeloma Clone

There are two types of largely mutually exclusive primary genetic events that give rise to the MM clone, each accounting for roughly half of myeloma cases: (1) hyperdiploidy, hypothesized to result from chromosome segregation errors occurring during rapid germinal center proliferation, and (2) IgH translocation, which is a result of the error-prone class switch recombination (CSR) process that activated B cells undergo [11]. Importantly, data suggests that MM is initiated by mutations associated with T cell-dependent B-cell activation [11]. T cell-dependent B-cell activation results in the activation of the co-stimulatory CD40-CD40L pathway within B cells [12,13], as well as release of T-cell cytokines IL4, IL21, and IL6 [14,15,16]. IL6 is a well-known activator of B cells and potent stimulator of B cell, plasma cell, and MM proliferation and survival [17]. B cells that undergo T cell-dependent activation rapidly proliferate and form germinal centers within lymphoid organs [18,19]. Within these germinal centers, activated B cells undergo rounds of division whereby a large repertoire of high-affinity antibodies is made through CSR and somatic hypermutation (SHM) [20]. CSR requires the formation of double-strand breaks and can therefore give rise to IgH translocations when aberrant recombination with other genomic regions occur [11]. The MM clone then homes into the BM mediated by the interaction of the MM receptor CXCR4 with the chemokine SDF1α [21]. Within the BM microenvironment, MM interacts with the extracellular matrix (ECM), which consists of proteins such as fibronectin, collagen, osteopontin, hyaluronan, and laminin; and cellular compartments including the BM stromal cells (BMSCs) [7]. Direct contact of MM cells with BMSCs activates downstream pathways such as MAPK, NOTCH, and Pi3K, which results in the secretion of pro-survival cytokines [7]. Examples include IL6, which facilitates MM survival, proliferation, migration, and drug resistance through the MEK/MAPK, JAK/STAT, and PI3K/Akt pathways. Other cytokines include the B-cell activating factor (BAFF) and a proliferation inducing ligand (APRIL), both of which promote MM proliferation via MAPK and NFκB, TNFα, insulin-like growth factor (IGF), hepatocyte growth factor (HGF), basic fibroblast growth factor (bFGF), and vascular endothelial growth factor (VEGF) [7]. Progression from MGUS to MM is then a multistep complex process involving further acquisition of secondary genetic events that confer a selection advantage, as well as immune modifications in the tumor microenvironment [22].

## 3. The Role of the Immunosuppressive Microenvironment in the Progression of MM

One key step in tumorigenesis is the ability of cancer to evade immune surveillance, the process by which a competent immune system recognizes and eliminates tumor cells [23]. MM clones and subclones that harbor mutations that allow them to escape immune destruction are selected for growth within the BM microenvironment [24]. Additionally, MM cells maintain a highly immunosuppressive BM microenvironment by promoting the expansion of myeloid-derived suppressor cells (MDSCs), tumor-associated M2-like macrophages (M2 TAMs), N2 neutrophils, regulatory T cells (Tregs), regulatory B cells (Bregs), and plasmacytoid dendritic cells (pDCs) [8,25]. 

The immunosuppressive MM BM microenvironment consists of myeloid-derived suppressor cells (MDSCs), tumor-associated M2-like macrophages (M2 TAMs), N2 neutrophils, regulatory T cells (Tregs), regulatory B cells (Bregs), and plasmacytoid dendritic cells (pDCs) [8]. Multiple immunotherapeutic approaches have been developed including antibody-based therapies (e.g., monoclonal antibodies, bispecific antibodies) and cellular therapies (e.g., CAR T cells, CAR-NK cells, CAR-M) to target MM within the context of the BM. Despite high response rates in patients treated with multiple prior lines of therapy, one limitation to current immunotherapeutic approaches is the lack of durability and the high rates of relapse. Studies aimed at uncovering the mechanism of MM immune evasion and effector cell exhaustion are currently underway.

### 3.1. Regulatory T Cells (Tregs)

Tregs are a subset of CD4^+^ T cells that express forkhead box P3 (FoxP3) and function to suppress immune response to maintain self-tolerance [26]. FoxP3 is the main transcription factor enabling the function of Tregs, and Tregs are immunophenotypically characterized by CD3^+^CD4^+^CD25^hi^CD127^lo^FoxP3^+^ [27]. In the context of the immunosuppressive MM BM microenvironment, Tregs inhibit MM-specific T-cell effector functions through direct cell-to-cell contact and the secretion of IL10, TGFβ, and IL35, as well as cytolytic granzymes and perforins, to inhibit and/or kill immune effector cells [28,29,30]. TGFβ is not only immunosuppressive but under the right conditions and together with IL2, has been shown to be able to induce the expression of FoxP3 in effector cells resulting in their functional conversion to Tregs [31]. In vitro studies have also shown that direct contact with MM cells is able to induce the development of CD4^+^CD25^+^FoxP3^+^ Tregs from an initial population of CD4^+^CD25^−^FoxP3^−^ cells, mediated by an inducible T-cell co-stimulator (ICOS) on T cells and their ligands (ICOS-L) expressed on MM cells [32]. Notably, one study showed that a lenalidomide and dexamethasone combination is able to reduce ICOS-L expression on MM cells and inhibit Treg differentiation measured by decreased FoxP3 expression [33]. However, other studies have reported conflicting results that lenalidomide increases Treg frequency in patients with MM and chronic lymphocytic leukemia [34,35,36,37]. This discrepancy in response to lenalidomide treatment may be dose- and time-dependent where short-term treatment induces Treg but prolonged treatment with increasing doses of lenalidomide inhibits Tregs [34,35]. Another potential explanation could be due to IMiD’s ability to downregulate CXCR4 expression on Tregs [38]. CXCR4-expressing Tregs are attracted to the MM BM microenvironment by cytokines such as stromal-derived-factor-1ɑ (SDF-1α), CXCR4 ligand [38]. Downregulation of CXCR4 may therefore allow Tregs to leave the BM and accumulate within the peripheral blood. Other anti-MM agents such as dexamethasone, cyclophosphamide, and daratumumab have also been shown to induce Treg apoptosis [33,39,40,41,42,43,44].

Indoleamine (IDO) is a cytosolic enzyme that degrades tryptophan into kynurenine (KYN). IDO-mediated KYN production promotes Treg development, stabilization, and activation, while suppressing effector T cells [45,46]. KYN is overexpressed in the serum and bone marrow of patients with MM (compared to healthy controls) and also higher in patients with ISS stage II/III disease (compared to ISS stage I) [46]. IDO was found to be expressed by CD138^+^ MM cells, as well as MM patient-derived BMSCs that were stimulated with IFNγ [46]. High IDO and KYN expression correlated with increased Treg accumulation with the MM BM microenvironment [46]. Conversely, inhibition of IDO with D,L-1-methyl-tryptophan inhibited MM-mediated Treg expansion and promoted T helper type 1 (Th1) differentiation [46]. Engagement of programmed cell death 1 (PD1) on the surface of Th1 cells by PD1 ligand (PDL1)-overexpressing cells or coated beads can also induce Tregs in vitro [47,48]. A proliferation-inducing ligand (APRIL), which is highly secreted by myeloid precursor cells (e.g., megakaryocytes, eosinophils, monocytes) and osteoclasts within the MM BM microenvironment [49], canonically binds B-cell maturation antigen (BCMA) on the MM-cell surface to drive survival and proliferation, has also been shown to bind a transmembrane activator, calcium modulator, and cyclophilin ligand interactor (TACI) on Tregs to drive MM-mediated Treg differentiation, proliferation, and survival [50,51].

The role of Tregs in MM pathogenesis and progression remains unclear, and conflicting studies have shown increased [29,52,53,54,55], decreased [36,56], or unchanged [57,58] Treg frequency in patients with MM. This lack of consistency is likely due to differences in the compartment studied (peripheral blood vs bone marrow), different immunophenotypic definitions of Tregs (CD4^+^FoxP3^+^, CD4^+^CD25^hi^, CD4^+^CD25^+^FoxP3^+^, CD4^+^CD25^+^CD127^−^), and interpatient tumor heterogeneity. The compartment matters as one study showed that Tregs are enriched in the peripheral blood (but not BM) of patients with treatment-naïve MM when compared to MGUS and controls [59]. The same study, however, did show that activated and memory Tregs were enriched in both the peripheral blood and BM of patients with treatment-naïve MM but that resting Tregs were significantly higher in controls [59]. Although this corresponded with increased terminally differentiated CD8^+^ effector cells seen in patients with treatment-naïve MM and increased central memory CD8^+^ T cells in controls, the study did not show a difference in the in vitro suppressive capacity of Tregs isolated from treatment-naïve MM compared to controls [59]. Specifically, Tregs were isolated from MM and controls and co-cultured with CD3/CD28 bead-activated PBMCs, and flow cytometry was used to analyze markers of activated T cells (i.e., CD69 and CD154 expression on CD4^+^ and CD8^+^ T cells). MM-derived Tregs did not significantly decrease the proportion of CD69 or CD154-expressing activated T cells when compared with healthy donor-derived Tregs [59].

Finally, studies comparing the frequency of Tregs in the BM versus peripheral blood of patients with MM have also reported inconsistent results, with some studies reporting higher frequencies in the MM BM [59,60,61] and others reporting similar peripheral blood and BM Treg frequencies [52,55].

### 3.2. Plasmacytoid Dendritic Cells (pDCs)

Plasmacytoid dendritic cells (pDCs) are a specialized subset of CD123 and CD303 co-expressing dendritic cells derived from BM hematopoietic stem cells [62]. Physiologically, pDCs are part of innate immunity and regulate anti-viral responses [62]. Upon stimulation by viral RNA and DNA, pDCs produce IFNγ and differentiate into professional antigen-presenting cells to stimulate T cells of the adaptive immune system [62]. pDCs eventually leave the BM and migrate directly into primary lymphoid organs and T cell-rich areas of secondary lymphoid tissues where they reside normally [62]. However, it has been reported in a number of human tumors that malignant cells recruit pDCs to the tumor microenvironment to help with immune tolerance [63]. This preferential accumulation of pDCs within the BM (as opposed to peripheral blood) is similarly seen in MM [64]. Compared to healthy donor-derived BM samples, MM patient-derived BM samples had higher frequencies of pDCs, and while no significant difference in pDC number was observed between healthy BM vs peripheral blood, an increased number of pDCs were noted in MM BM vs MM peripheral blood [64]. Importantly, in vitro co-cultures show that pDCs isolated from MM BM were able to induce proliferation and confer bortezomib resistance in MM cell lines [64]. Additionally, pDCs isolated from the MM BM showed significantly impaired ability to trigger T-cell proliferation (assessed by 3H-thymidine incorporation assay) when compared to normal pDCs [64].

pDCs highly express PDL1, which is further enhanced by pDC-MM interaction, and this leads to inhibition of T- and NK-cell function [65]. Consistent with this, blockade of the PDL1-PD1 interaction with the anti-PDL1 antibody was able to restore MM-specific cytotoxic T-cell and NK-cell activity [65]. MM-derived pDCs were also able to trigger MM proliferation and confer drug resistance to bortezomib in vitro [64]. Mechanistically, pDC-MM contact stimulates the secretion of IL10, VEGF, CD40L, IL8, IL15, IL6, and MCP1 [64]. This growth-promoting effect can be abrogated by disrupting NF-κB through the inhibition of the B-cell activating factor (BAFF)/APRIL and receptor activator of NF-κB (RANK)/RANKL signaling [64]. Additionally, because pDCs strongly expresses CD38, daratumumab induces strong depletion of pDCs [66].

### 3.3. Myeloid-Derived Suppressor Cells (MDSCs)

Myeloid-derived suppressor cells (MDSCs) are a heterogeneous population of immature myeloid cells that have been shown to accumulate in both the peripheral blood and BM of patients with MM [67]. There are two types of MDSCs: monocytic MDSC (M-MDSC) and polymorphonuclear MDSC (PMN-MDSC) that are distinguished by CD14 expression [68]. M-MDSCs (CD11b^+^**CD14^+^**CD33^+^HLA-DR^low/−^), in particular, are enriched in patients with newly diagnosed and relapsed MM compared with patients in remission, and high levels of M-MDSCs have been found to correlate with MM progression and treatment resistance [69]. Functionally, MDSCs induce T-cell apoptosis by producing nitric oxide and suppress T-cell function by producing reactive oxygen and nitrogen species, as well as deplete the microenvironment of L-arginine and L-cysteine, which are used to produce the CD3ζ-chain, a component of the T-cell receptor [70,71,72,73].

In vitro co-culture experiments show that exposure of peripheral blood mononuclear cells (PBMCs) obtained from healthy donors to direct contact with MM cells, conditioned media from MM cell lines, and plasma from newly diagnosed patients can induce the development of MDSCs [69,74,75]. The C-C motif chemokine ligand 5 (CCL5) and macrophage migration inhibitory factor (MIF) have recently been found to be key soluble mediators of MDSC induction and are secreted by MM cells [76]. The same study found that both lenalidomide and pomalidomide were able to decrease MM expression of CCL5 (through cereblon-dependent pathways) and MIF (through cereblon-independent pathways) [76]. Both IMiDs were also able to decrease expression of the C-C motif chemokine receptor 5 (CCR5) and induce interferon regulatory factor 8 (IRF8) expression, a negative regulator of differentiation towards MDSCs [76].

### 3.4. Cancer-Associated Fibroblasts (CAFs)

Cancer-associated fibroblasts (CAFs) are a heterogeneous population of CD45^−^ BM stromal cells expressing different levels of fibroblast specific protein 1 (FSP1), alpha smooth muscle actin (αSma), and fibroblast activating protein (FAP) [77,78]. CAFs have recently been shown to mediate MM proliferation and therapeutic resistance through the production of various cytokines (e.g., IL6, TGFβ), chemokines (e.g., SDF-1), and pro-inflammatory and pro-angiogenic factors (e.g., VEGF) [77,78]. Consistent with this, BM CAFs are enriched in patients with newly-diagnosed and relapsed MM compared to patients with MGUS or non-MM controls [78]. In vivo studies with syngeneic 5T33MM and xenograft mouse models show that MM cells are able to induce CAF proliferation [78]. Direct contact between MM cells and CAFs through CXCL12/CXCR4 and other integrins is necessary to facilitate the tumor-promoting functions of CAFs [77]. Strategies aimed at depleting CAFs using monoclonal antibodies and CAR T cells are currently being studied preclinically and will be discussed later.

### 3.5. Tumor-Associated M2-like Macrophages (M2 TAMs)

Mature macrophages are identified by the surface markers CD16, CD68, CD115, CD163, and CD312, and represent a major aspect of the innate immune system [79]. Beyond phagocytosis of pathogens and apoptotic cells, macrophages have both tumor promoting and tumor killing effects depending on environmental cues [80]. Two flavors of macrophages exist depending on function: (1) M1 macrophages, which are classically activated by interferon-γ (IFNγ) or lipopolysaccharide (LPS), facilitate tumor killing through phagocytosis, the release of nitric oxide and reactive oxygen species, as well as proinflammatory cytokines (i.e., IL1, IL6, IL8, IL12, TNFα) [80,81,82], and (2) the IL4 dependent alternatively-activated M2 macrophages, which are anti-inflammatory [83,84]. M2 macrophages support tumor growth by secreting vascular endothelial growth factor (VEGF), transforming growth factor-β (TGFβ), IL10, and also through expression of PD-L1 [83,84,85].

Phenotypically and functionally similar to M2 macrophages are CD163 and CD206 expressing tumor-associated M2-like macrophages (M2 TAMs) [86]. TAMs originate from peripheral blood monocytes that infiltrate into the tumor microenvironment in response to cytokines such as VEGF, colony stimulating factor-1 (CSF1), CXC motif chemokine ligand-12 (CXCL12) and various CC motif chemokine ligands (CCL) such as CCL2 [87,88]. TAMs make up about 10% of the BM of patients and have been found in higher proportions in patients with aggressive disease [89,90,91]. In vitro studies have recently shown that the IL10 secretion by MM cells polarized macrophages towards M2 phenotype and that inhibition of IL10 signaling with an IL10 receptor blocking antibody resulted in reversal of the M2 phenotype and loss of TAM-mediated MM proliferation and drug resistance [92]. Functionally, TAMs support MM proliferation (by secreting IL6), angiogenesis (by secreting VEGF, CCLs, matrix metalloproteinases (MMPs)), and immunosuppression (by secreting IL10, TGFβ, indoleamine 2,3-dioxygenase (IDO) and inhibiting IL12 and TNFα production) in the tumor microenvironment [93,94].

Other strategies to target TAMs in the MM BM microenvironment include (1) depleting TAMs by direct killing with clodronate-liposome or inhibiting chemokine signaling (e.g., CXCL12-CXCR4, CCL2-CCR2 to interrupt monocyte recruitment into the BM [95,96,97,98,99], (2) polarizing TAMs towards an M1 phenotype by inhibiting CSF1 receptor signaling or the pro-M2 cytokine macrophage migration inhibitory factor, or by using granulocyte-macrophage CSF (GM-CSF) or the Janus kinase (JAK) 1/2 inhibitor ruxolitinib, both of which have been shown to induce M1 polarization [100,101], and (3) inhibiting the immunosuppressive effects of TAMs [79]. To this end, research into inhibiting IDO to restore T-cell function is currently underway [46].

### 3.6. N2 Neutrophils

Owing to significant phenotypic and functional overlap between N2 neutrophils and PMN-MDSCs, there has been some confusion on the classification of these cells [102]. In fact, density gradient centrifugation is the only way to phenotypically differentiate TANs from PMN-MDSCs (which end up on the low-density layer) [103]. Unlike T and NK cells, the role of neutrophils in the tumor microenvironment is less clear as tumor-associated neutrophils have been found to possess both anti-tumor (e.g., direct cytotoxicity and inhibition of metastasis) and pro-tumor activity (e.g., promote angiogenesis, stimulate tumor cell migration and invasion, contribute to immunosuppression) [102]. Similar to TAMs, neutrophils maintain functional plasticity and can be alternatively activated in response to microenvironmental cues. Specifically, the presence of TGFβ within the microenvironment polarizes neutrophils towards a protumor phenotype (termed N2 neutrophils), while the presence of interferon-β (IFNβ) results in an antitumor phenotype (termed N1 neutrophils) [102].

Neutrophils isolated from the peripheral blood of patients with newly diagnosed MM have been shown to have a different gene expression profile compared with healthy donor-derived neutrophils [104]. Compared with both healthy donors and MGUS, neutrophils derived from patients with MM had genes dysregulated in FC-γ-R mediated phagocytosis, endocytosis, leukocyte transendothelial migration, chemokine signaling Toll-like receptor pathways, and inositol-phosphate metabolism [104]. Functionally, there is limited data showing that neutrophils derived from both the peripheral blood and BM of patients with MM were able to inhibit T-cell proliferation in a similar fashion to PMN-MDSCs by producing ROS and/or arginase-1 (which depletes L-arginine from the microenvironment) [104,105]. Noteworthy, studies present conflicting results with some showing that neutrophils derived from the peripheral blood of patients with MM did not have an increased T-cell inhibitory effect compared with peripheral blood neutrophils from healthy donors [105].

### 3.7. Regulatory B Cells (Bregs)

Bregs are a subset of B cells characterized by CD19^+^CD24^high^CD38^high^ that, similar to Tregs, secrete IL10, IL35, and TGFβ and have been implicated in MM progression [106,107,108]. Bregs have been found to preferentially accumulate in the BM (as opposed to peripheral blood) of patients with MM [107]. Consistent with this, Bregs isolated from the BM of patients with MM were dependent on MM cells for survival in vitro as removal of CD138+ MM cells from the BM mononuclear cell culture resulted in Breg apoptosis as measured by the annexin V/propidium iodide apoptosis assay [107]. MM-derived Bregs were found to highly express TACI and the addition of APRIL in vitro increased the frequency of IL10-producing Bregs [50]. Functionally, a greater percentage of Bregs isolated from the BM were IL10 producing when compared to peripheral blood Bregs, and BM Bregs were able to inhibit elotuzumab associated antibody-dependent cellular cytotoxicity (ADCC) by NK cells [107].

Bregs also express PDL1 and CD1d on the cell surface, the latter of which is a nonpolymorphic MHC class I-like molecule found on antigen-presenting cells that facilitates the presentation of glycolipid antigens to natural killer T (NKT) cells, a process that is essential for the development of invariant NKT cells [106]. CD1d is also highly expressed by MM cells, especially during early stages of disease progression and MM cells can “hijack” this normally proinflammatory antigen presenting system to instead disrupt iNKT cell function [109]. MM cells secrete GM3 ganglioside, which together with the high levels of CD1d expression in the BM microenvironment, results in the formation of the CD1d/GM3 complex, which binds to the invariant T-cell receptor of iNKT resulting in iNKT deregulation, loss of IFNγ secretion, and immune evasion [109].

### 3.8. Impaired Immune Effector Killing in MM

The functional sequelae of the immunosuppressive BM microenvironment are diminished effector T-cell survival, proliferation, and function, evidenced by multiple CD4 and CD8 T-cell signaling defects (e.g., downregulation of CD28, CD152, CD3ζ, p56lck, ZAP-70, and PI3K; upregulation of exhaustion markers) in patients with advanced stage MM [110]. Additionally, the MM BM microenvironment is enriched for coinhibitory molecules (e.g., PDL1 on MM cells, Galectin-3 and HLA-DR on MM cells, Galectin-9 in the MM BM plasma; and corresponding immune checkpoint receptors PD1, LAG3, and TIM3 on T cells) [111,112,113,114,115,116,117,118]. Soluble factors such as IL6 (via JAK2/STAT3 and MEK/ERK signaling), IFNγ (via MyD88/TRAF6 and MEK/ERK signaling), and APRIL (via BCMA binding and MEK/ERK signaling) are found in high abundance within the MM BM microenvironment and have been implicated in promoting PDL1 expression [113,114,115,119]. In spite of this, clinical trials studying monotherapy with PD1/PDL1 inhibitors have yielded lackluster results, and this is likely due to the highly dysfunctional MM T cells, which coexpress T-cell immunoglobulin mucin-3 (TIM3) and lymphocyte-activation gene 3 (LAG3), and a combined blockade of multiple checkpoint pathways may be needed [116,117,120].

## 4. The Era of Immunotherapy in MM

Cancer immunotherapy is a treatment that enhances the body’s immune system to prevent, control, and kill cancer cells. With increasing clinical use and experience, the advantages of immunotherapy over traditional anti-tumor therapy are apparent. These include the potential for higher specificity, potency, applicability (as killing does not hinge on the presence of specific mutations), and persistence, with the additional benefit of more tolerable side effects [121]. Several immunotherapies are already in routine clinical use with many others currently under active investigation. Bolstered by unprecedented clinical efficacy, we are starting to see a shift in focus away from molecularly targeted therapy towards developing immunotherapies for MM.

## 5. Chimeric Antigen Receptor T (CAR T)-Cell Therapy

Chimeric antigen receptor T (CAR T)-cell therapy, and in particular CD-19 CAR T-cells, has recently been proven to be highly effective in the treatment of acute lymphoblastic leukemia (ALL) and non-Hodgkin lymphoma (NHL) (e.g., tisagenlecleucel/Kymriah, axicabtagene ciloleucel/Yescarta, lisocabtagene/Breyanzi, brexucabtagene/Tecartus) [122,123,124,125,126]. Compared with ALL and NHL, CAR T research in MM is still in the early stages with the first MM-specific BCMA CAR developed less than a decade ago [127]. A summary of CAR T-cell trials is detailed in Table 1. There are currently two FDA-approved BCMA-CARs for patients with heavily pretreated relapsed or refractory MM: idecabtagene/Abecma, which showed an overall response rate (ORR) of 73% with median progression free survival (PFS) of 12.1 months at target dose of 450 × 10^6^, and ciltacabtagene/Carvykti (bi-epitope CAR containing 2 BCMA-targeting domains), which had an ORR of 97% and median PFS was not reached (Table 1) [128,129]. However, unlike CAR T-cell therapy for ALL or diffuse large B-cell lymphoma (DLBCL), which has the potential to induce durable remissions, the majority of patients with MM treated with BCMA-CARs relapse within two years despite favorable response rates [130].

### 5.1. Mechanisms of Resistance to CAR T-Cell Therapy

Several mechanisms of CAR T-cell resistance were proposed including BCMA antigen escape and heterogeneity, as well as short-term T-cell persistence due to activation-induced cell death (AICD), poor T-cell expansion, and terminal effector T-cell differentiation and exhaustion [131,132]. Intra-tumoral BCMA heterogeneity can lead to the selection and proliferation of clones with low/no BCMA expression as MM cells with high BCMA expression are preferentially killed [133,134,135]. This is consistent with studies reporting loss of BCMA upon disease relapse after the first CAR T-cell infusion [136,137,138,139]. Antigen escape can occur because of permanent antigen loss from alternative splicing or heterozygous deletion of chromosome 16p and concomitant frameshift or missense mutation of the other allele [138,140,141], or reversible antigen loss through trogocytosis (active transfer of target antigen to T cells), increasing the risk of T-cell fratricide and exhaustion [142,143,144]. Additionally, BCMA is actively cleaved from the MM-cell surface by the ubiquitous γ-secretase (GS) complex, which not only decreases the density of antigen on MM cells but also releases a soluble BCMA (sBCMA) fragment that can act as a decoy to inhibit CAR T-cell function [145]. Conversely, small-molecule γ-secretase inhibitors (GSIs) have been shown to stabilize BCMA expression on the cell surface and decrease sBCMA, as well as enhance efficacy of CAR T-cell therapy; a phase I clinical trial (NCT03502577) investigating the combination of GSIs with concurrent BCMA CAR T-cell therapy is currently underway [145]. Notably, epigenetic modulation with all-trans retinoic acid (ATRA) increases BCMA expression on MM cells to improve recognition by BCMA CAR T cells [146]. A combination of ATRA and GSIs showed a synergistic effect compared with a single-agent treatment alone [146].

Another strategy to overcome antigen escape is to employ CAR T cells that concomitantly target multiple antigens using either dual CAR constructs or tandem CARs (i.e., a single CAR construct expressing two single chain variable fragments/scFVs) [147]. Several dual CAR T-cells targeting BCMA/CD19 (NCT04162353), BCMA/SLAMF7 (NCT04156269), BCMA/CD38 (NCT03767751), BCMA/NY-ESO1 (NCT03638206), and CD38/CD19 (NCT03125577) are currently being evaluated in clinical trials (NCT03271632, NCT03473496) (Table 1) [147]. Dual targeting cancer-associated fibroblasts (CAFs) and MM cells is another novel strategy aimed at overcoming CAR T-cell resistance that has demonstrated promising preclinical results [148]. In one study, Sakemura et al. first showed that CAFs isolated from the BM of patients with MM significantly enhanced MM tumor growth both in vitro and in vivo, potently suppressed BCMA CAR T-cell expansion and CAR T-cell degranulation, and significantly altered the CAR T-cell cytokine profile (increased inhibitory cytokines and growth factors: TGFβ, fibroblast growth factor 2 (FGF2), growth-regulated oncogene, IL4, IL5, MCP1, MCP2; decreased effector cytokines: IFNγ, GM-CSF, soluble CD40L, TNFα, TNFβ, macrophage inflammatory protein 1β) [148]. The BM-CAFs were shown to highly express FAP and the signaling lymphocyte activation molecule family-7 (SLAMF7) [148]. The authors then generated dual CAR T-cells targeting MM (via BCMA), as well as CAFs (via either FAP or SLAMF7) and found that both BCMA-FAP and BCMA-SLAMF7 dual targeting CARs were able to overcome CAF-induced CAR T-cell inhibition in an MM-tumor microenvironment (TME) mouse model where the NOD/SCID γ chain-deficient (NSG) mice were simultaneously intravenously injected with both the OPM-2 MM cell line and BM-derived CAFs [148].

Various T-cell directed strategies have emerged to improve CAR T-cell persistence and potency, and these include (1) refinement of the CAR construct to reduce immune rejection, improved binding affinity, and prevention of ligand-independent tonic signaling, (2) development of fourth-generation armored CAR T cells to overcome the immunosuppressive MM BM microenvironment, (3) optimizing T-cell subpopulations prior to CAR T-cell reinfusion (e.g., adjusting the CD4^+^/CD8^+^ ratio to 1:1; NCT03430011 and NCT03338972; addition of the PI3K inhibitor bb007 during ex-vivo culture to enrich for memory-like T cells and decrease the proportion of terminally differentiated or senescent T cells; NCT03274219), and (4) using BM-derived T cells (i.e., marrow-infiltrating lymphocytes), which display enhanced memory phenotype and long-term persistence, or allogeneic lymphocytes (i.e., off-the-shelf CAR T cells), which represent a readily available source of T cells without prior exposure to anti-MM therapy [149]. One method to reduce the autoreactivity of allogeneic “off-the-shelf” CAR T cells involves the use of the CRISPR/Cas9 system to knockout the T-cell receptor and MHC class I, coupled with specific insertion of the CAR at the TRAC locus (NCT04244656) [150].

Various microenvironmental-directed strategies to improve CAR T-cell therapy are also currently being investigated, and these include (1) optimization of the dose, timing, and lymphodepleting conditioning regimen used to eliminate immunosuppressive cells and reduce the pool of cells competing for homeostatic cytokines, (2) incorporating CAR T-cell therapy earlier in the disease course prior to adverse changes in the immune microenvironment, and (3) combination therapy with other therapies such as IMiDs and immune checkpoint inhibitors [149].

### 5.2. Exploiting the Tumor Microenvironment to Overcome CAR T-Cell Therapy-Related Toxicities

Further problems limiting CAR T-cell therapy include toxicity from uncontrolled systemic cytokine levels resulting in cytokine release syndrome (CRS), hemophagocytic lymphohistiocytosis (HLH) and/or macrophage activation syndrome (MAS), and immune effector cell-associated neurotoxicity syndrome (ICANS), as well as on-target/off-tumor effects resulting in B-cell aplasia, neutropenia, and immunosuppression leading to an increased risk of infections [132]. Current treatment of CRS and ICANs is largely supportive with the addition of corticosteroids ± tocilizumab or siltuximab (IL6 antagonists) in severe cases [132]. Treatment of HLH/MAS may require the addition of systemic etoposide or intrathecal cytarabine (for neurotoxicity) in cases refractory to corticosteroids and IL6 antagonism [132]. Other strategies to overcome systemic cytokine toxicities of CAR T cells include engineering a suicide receptor (e.g., CD20 or truncated EGFR) on the CAR T cell, which can then be eliminated by antibody-dependent cellular cytotoxicity (ADCC) or complement-dependent cytotoxicity (CDC)-inducing monoclonal antibodies (e.g., rituximab or cetuximab), as well as engineering CAR T cells that secrete factors to neutralize the pro-inflammatory cytokines (e.g., anti-GM-CSF monoclonal antibody, IL1 receptor antagonists) or knock out cytokine genes [132].

### 5.3. Beyond CAR T Cells (CAR-NK and CAR-Macrophages)

Natural killer (NK) cells are the main anti-tumor effector cells in innate immunity and are also key mediators of ADCC [151]. More recently, adoptive transfer of NK cells has been implemented in the treatment of certain types of cancer including acute myeloid leukemia, ovarian, and breast cancer [151]. In post-ASCT MM patients, a clinical trial studying the use of ex vivo activated and expanded autologous NK cells as consolidation therapy showed NK-cell persistence in the circulation and increased granzyme B levels within the BM up to 4 weeks post-infusion [152]. The study also reported reduction in M-component and/or deepening of minimal residual disease post NK infusion [152]. Bolstered by the success of CAR T-cell therapy, research on the engineering of CAR-NK cells against MM is currently well underway; and preclinical data on CAR-NK against NKG2D ligands, CD38, and BCMA demonstrate potent and specific anti-MM cytotoxicity [153,154,155]. Compared with CAR T cells, CAR-NK cells have a number of unique advantages, including (1) minimal risk of CRS and neurotoxicity in the autologous setting and graft versus host disease in the allogeneic setting, (2) potential ease of “off-the-shelf” manufacturing owing to reduced risk of alloreactivity, and (3) the ability to natively kill tumor cells through CAR-independent mechanisms such as CD16-mediated ADCC, natural cytotoxicity receptor-mediated and/or NKG2D-mediated ligand binding [156].

Macrophages have historically been highly resistant to genetic engineering with standard lentiviral, retroviral, and adenoviral vectors, which have limited our ability to exploit them as antitumor therapeutics [157]. The group at the University of Pennsylvania recently found that chimeric adenoviral vector Ad5f35 was able to transduce primary human monocytes and macrophages with high efficiency and reproducibility. Utilizing this system, they were able to engineer CAR macrophages (CAR-Ms) [157]. CAR-Ms displayed antigen-specific phagocytosis and tumor eradication both in vitro and in two solid tumor xenograft mouse models. Notably, the CAR engineering process also polarized the macrophages towards a sustained pro-inflammatory (M1) phenotype [158]. Functional characterization showed that CAR-Ms expressed pro-inflammatory cytokine and chemokine, upregulated antigen presentation machinery, and converted bystander M2 macrophages to M1, as well as boosted anti-tumor T-cell activity [158].

### 5.4. Bispecific T-Cell Engagers (BiTEs)

Bispecific antibodies (BsAbs) are antibodies with two binding sites that recognize two different antigens or two different epitopes on the same antigen [159]. Three categories of BsAbs exist based on their targets: antibodies targeting (1) two different tumor antigens, (2) one tumor antigen and one immune-related molecule, and (3) two immune-related molecules [160]. Bispecific T cell-recruiting antibodies, which belong to the second category, have recently garnered a lot of interest for their ability to target tumor-associated antigens and CD3 to redirect autologous T cells against cancer cells [160]. Bispecific T-cell engagers (BiTEs) are the most well-established format of variable fragment (Fv)-based bispecific T cell-recruiting antibodies and they consist of two single-chain variable fragments (scFvs) connected by a short peptide linker. Other Fv-based formats include dual-affinity retargeting antibodies (DARTs), tandem diabody (TandAb), and single-chain diabody [160]. BiTEs require the presence of target tumor cells to induce activation and proliferation of T cells [160]. The activated T cells secrete cytolytic granzymes, which lead to tumor-cell lysis [160]. Notably, BiTEs are able to activate memory T cells without costimulatory signals such as CD28 and IL2 and can even overcome T-cell exhaustion [160]. Unlike CAR T cells, BiTEs are available off-the-shelf and do not require a prerequisite number of donor leukocytes/lymphocytes, or the expensive, time-consuming, and error-prone CAR T-cell manufacturing process [161]. Additionally, BiTEs are, in and of themselves, not living products and have relatively short half-lives so treatment can be stopped at any time to reverse immune activation and immune-related adverse events [161].

The predominant target for MM BiTE studies is BCMA, with a few studies looking at CD19, CD38, GPRC5D, and FcRH5 (Table 2). Preliminary results from eight BCMA BiTE phase 1 or 1/2 trials in heavily pretreated RRMM are very promising, boasting response rates ranging from 52–83% with minimal grade ≥3 CRS or neurotoxicity (Table 2) [162,163]. Durability of BiTEs in MM is still under investigation but preclinical studies show rapid relapse after treatment discontinuation in de novo VK*MYC mice (model of NDMM) and transient responses with relapse occurring within 3 weeks of treatment in the more aggressive transplantable VK12598 syngeneic VK*MYC model [164]. Relapse was mainly seen in mice with high initial tumor burden, and associated with loss of T-cell functionality, suggesting that high levels of tumor antigen may have the detrimental effect of overactivating T cells and may not be beneficial in vivo [164]. Notably, cyclophosphamide preconditioning was able to induce long-lasting remission in both models of MM, even after tumor rechallenge [164]. This was due to cyclophosphamide’s ability to preferentially kill Tregs and the cytoreductive effect, which tempered the activation of T cells and prevented BiTE-induced T-cell exhaustion [164]. Another method to counteract T-cell exhaustion resulting from persistent antigen stimulation or tonic receptor signaling would be to incorporate treatment-free intervals to functionally reinvigorate T cells [165].

### 5.5. Beyond BiTEs (Bispecific Killer Cell Engagers, Trispecific Antibodies, and BsAbs Armed T-Cell Therapy)

More recently, bispecific killer cell engagers (BiKEs) have been developed to harness the cytotoxic ability of NK cells. Instead of binding to CD3 on T cells, BiKEs recognize CD16, NKp30, or NKG2D on NK cells [166]. A number of preclinical BiKEs (e.g., BCMA × CD16A, BCMA × MICA, BCMA × NKp30, CS1-NKG2D) have demonstrated promising MM-specific cytotoxicity [167,168,169,170]. Trispecific antibodies are also currently in preclinical investigation which, in addition to a T-cell binding domain and distinct myeloma antigen binding domain, includes a third domain that binds another distinct myeloma antigen or adds either T-cell costimulatory proteins to decrease T-cell anergy or cytokines to promote effector cell activation (e.g., IL-15 containing TriKEs) [170]. An example of trispecific killer engagers (TriKEs) being studied in MM is the dual antigen targeting BCMA × CD200 × CD16A antibody called “aTriFlex” [171].

Another novel adoptive T-cell transfer-based immunotherapy that combines the advantages of CAR T cells and BiTEs is BsAbs armed T-cell therapy (BAT). After leukapheresis, leukocytes are coated with BsAbs ex vivo prior to reinfusion, which has shown favorable anti-tumor effects and allows for more precise control of potency compared with CAR T-cell therapy as the amount of BsAbs used to arm the T cells can be controlled, as can the cell dose per infusion and number of infusions [172].

## 6. Monoclonal Antibodies (MAbs)

### 6.1. CD38 MAbs

Daratumumab is a first-in-class CD38 receptor targeting IgG1k mAb. CD38 is overexpressed on MM cells but is also present at lower levels on normal plasma cells, and myeloid and lymphoid cells (including NK cells), as well as red blood cells and platelets [173,174]. By binding to CD38, daratumumab promotes MM-cell apoptosis via antibody-dependent cellular cytotoxicity (ADCC), complement-dependent cytotoxicity (CDC), inhibition of mitochondrial transfer, Fc receptor-mediated cross-linking, and antibody-dependent cellular phagocytosis [175,176]. Notably, IMiDs such as lenalidomide and pomalidomide are able to potentiate NK cell-mediated ADCC by increasing the expression of NK-cell activating ligands MICA and PVR/CD155 on MM cells, which facilitates tumor recognition and killing by NK cells [177]. Because CD38 is not exclusively expressed MM cells, off-target effects include NK depletion via cell fratricide as well as immunoparesis (defined as the reduction in levels of ≥1 immunoglobulins not associated with the patient’s specific myeloma variant) resulting from depletion of the antibody-producing plasma/B-cell population [178,179]. This is especially important given the COVID-19 pandemic we are currently in, and studies showing that MM patients mount a poor humoral response following vaccination, especially during treatment with anti-CD38 therapy [180]. One favorable on-target/off-tumor effect of CD38 mAbs is their ability to deplete immunosuppressive cells such as Tregs and Bregs [42,43].

Daratumumab was the first monoclonal antibody approved by the FDA for treatment of MM (Table 3). This occurred on November 16, 2015 following results from the SIRIUS trial, and specifically approved its use for patients with multiple myeloma who have received at least three prior lines of therapy, including a proteasome inhibitor (PI) and an immunomodulatory agent, or those patients who are double refractory to a PI and immunomodulatory agent. In the SIRIUS trial, daratumumab monotherapy for this patient population provided a 3.7-month median progression free survival (PFS) and overall response rate (ORR) of 29.2%. The following year, in November 2016, based on the results of the POLLUX and CASTOR trials, daratumumab in combination with lenalidomide and dexamethasone (Dara-Rd) or daratumumab in combination with bortezomib and dexamethasone (Dara-Vd), respectively, were approved by the FDA for treatment of patients with MM who have received at least one prior therapy [181,182,183,184]. The most recent updates of the POLLUX and CASTOR studies showed median PFS of 44.5 and 16.7 months, respectively, when compared to Rd or Vd alone, at 17.5 months and 7.1 months, respectively [183,184]. By May 2018, based on the ALCYONE study, the FDA-approved daratumumab in combination with bortezomib, melphalan, and prednisone (Dara-VMP) for the treatment of newly diagnosed multiple myeloma (NDMM) in patients who were transplant ineligible [185]. Later, in September, 2019, the FDA also approved daratumumab in combination with bortezomib, thalidomide, and dexamethasone (Dara-VTd) for treatment of NDMM in transplant-eligible patients based on results from the CASSIOPEIA study [186]. Other pertinent trials include the MAIA trial that led to the 2019 FDA approval of Dara-Rd for upfront treatment of NDMM patients who are transplant ineligible [187]. The CANDOR and EQUULEUS studies helped lead to FDA approval of carfilzomib and daratumumab with dexamethasone for RRMM [188,189]. More recently, the APOLLO study examined daratumumab in combination with pomalidomide and carfilzomib for RRMM, with its results leading to FDA approval of this regimen in July 2021 [190].

Similar to daratumumab, isatuximab targets the CD38 receptor, but binds to a different CD38 epitope (Table 4). Mechanistically, the main way isatuximab induces MM apoptosis is through ADCC [191]. Unlike daratumumab, isatuximab had minimal CDC activity [191] but is able to directly induce MM apoptosis in the absence of cross-linking antibodies and independently of effector cells, even in p53 mutant MM [192]. In March 2020, the FDA-approved isatuximab in combination with pomalidomide and dexamethasone (IPd), based on results from the ICARIA-MM study, for the treatment of adults with RRMM who have received at least two prior therapies including lenalidomide and a proteasome inhibitor [193]. A year later, in March, 2021, the FDA also approved isatuximab in combination with carfilzomib and dexamethasone (IKd) for treatment of adults with RRMM who have received one to three prior lines of therapy based on results from the IKEMA study [194].

### 6.2. SLAMF7 MAbs

Elotuzumab is a humanized IgG kappa monoclonal antibody that targets the signaling lymphocyte activation molecule family member 7 (SLAMF7) to promote MM killing, predominantly through NK cell-mediated ADCC, as well as macrophage-mediated ADCP [195,196,197,198]. SLAMF7 (also known as CS1, CRACC, or CD319) is highly expressed in myeloma cells but is either less strongly or not expressed by normal hematopoietic stem cells [199]. SLAMF7 is also expressed on NK cells and elotuzumab is able to bind NK cells through and activate CD16/Fc receptor-independent costimulatory signaling to enhance NK cytotoxicity via the upregulation of NKG2D, ICAM-1, and activated LFA-1 [197]. Notably, although elotuzumab binds SLAMF7 on NK cells, preclinical in vitro studies as well as patient data from clinical trials indicate that, unlike daratumumab, elotuzumab does not induce significant NK cell fratricide [200]. SLAMF7 was also highly expressed on exhausted CD8^+^ T cells and CD8^+^CD28^−^CD57^+^ Tregs in patients with MM [201]. Consistent with this, gene expression profiling of SLAMF7-expressing CD8^+^ T cells revealed higher expression of exhaustion markers including LAG3, TNFRSF1B, CD244 (2B4), and TIM-3 compared with their SLAMF7-negative counterparts [201]. Elotuzumab was able to specifically eliminate the SLAMF7-expressing CD8^+^ T cells (including CD8^+^CD28^−^CD57^+^ Tregs) through ADCP but not ADCC [201]. Soluble SLAMF7 (sSLAMF7) has been found to be overexpressed in the sera of some patients with MM but not healthy donors [195]. sSLAMF7 has been implicated in MM proliferation via homophilic interaction with surface SLAMF7 and downstream activation of SHP-2 and ERK signaling [202]. Lenalidomide, through IKZF1 degradation, decreases expression of SLAMF7 and abrogates the growth-promoting effect of sSLAMF7 [202]. Notably, elotuzumab was also able to bind and neutralize sSLAMF7 to inhibit sSLAMF7-induced MM growth [202].

FDA approval for elotuzumab was first granted in November 2015 in combination with lenalidomide and dexamethasone (ERd) for treatment of patients with RRMM and who have received one to three prior medications (Table 5). These recommendations were based on results from the ELOQUENT-2 trial, which compared ERd versus Rd alone [203]. Both median PFS in months and ORR were improved in the ERd compared with the Rd cohort at 19.4 months vs. 14.9 months and 79% vs. 66%, respectively [203]. In November 2018, based on the ELOQUENT-3 study, the FDA then approved elotuzumab in combination with pomalidomide and dexamethasone (EPd) for the treatment of patients with RRMM who had received at least two prior therapies including lenalidomide and a proteasome inhibitor [204]. ELOQUENT-3 compared patients on EPd versus pomalidomide and dexamethasone (Pd) alone and found almost a doubling of both the median PFS and ORR [204].

### 6.3. Antibody Drug Conjugates (ADCs)

Antibody drug conjugates (ADCs) are a class of highly targeted biopharmaceutical drugs consisting of a potent cytotoxic compound chemically linked to a monoclonal antibody. ADC binding to a specific tumor-associated antigen results in internalization of the cytotoxic payload and cell death. ADCs, therefore, do not solely rely on the recruitment of effector cells or activation of complement cascade to kill MM cells [205].

Belantamab is the first-in-class ADC consisting of an anti–B-cell maturation antigen (BCMA)-directed antibody conjugated to monomethyl auristatin F (MMAF), a microtubule inhibitor [51]. Belantamab eliminates MM cells by (1) triggering cell-cycle arrest through MMAF inhibition of the microtubule network, (2) inhibiting BAFF/APRIL binding to BCMA and downstream NF-κB activation, and (3) enhancing ADCC [51]. In August 2020, the FDA-approved belantamab for treatment of adult patients with RRMM who had previously received at least four therapies, including an anti-CD38 monoclonal antibody, a proteasome inhibitor, and an immunomodulatory agent (Table 6) [206]. Approval was granted based on results from the DREAMM-2 study, a phase II, open-label, two-arm multicenter trial, which found an ORR of 31% (median PFS 5.7 months) for patients who received the recommended dose of 2.5 mg/kg of belantamab [206]. Examples of ADCs currently under clinical investigation include lorvotuzumab mertansine (anti-CD56 conjugated with maytansinoid) [207], milatuzumab doxorubicin (anti-CD74 conjugated to doxorubicin [208], and indatuximab ravtansine (anti-CD138 conjugated to maytansinoid) [209].

### 6.4. MAbs against Other Targets

Other targets for which mAbs are currently being developed are B-cell activating factor (BAFF; NCT01556438) [210], CD74 (NCT00421525) [211], and IL6 [212], as well as the immune checkpoint proteins programmed cell death 1 (PD1; NCT01592370) [213,214] and its ligand (PDL1; NCT02579863, NCT02431208) [215,216], cytotoxic T-lymphocyte-associated protein 4 (CTLA-4; NCT01592370) [217], lymphocyte-activation gene 3 (LAG3; NCT04150965), and T-cell immunoreceptor with Ig and ITIM domains (TIGIT; NCT04150965).

## 7. Conclusions and Future Perspectives

The therapeutic landscape in MM has evolved significantly since Melphalan was first used in 1958. Although the introduction of novel therapies such as proteasome inhibitors, IMiDs, have led to great improvement in patient outcomes, these agents are eventually cleared from the body over time, making it difficult to achieve durable responses without continual administration. In fact, most patients still relapse even when these therapies are used in maintenance regimens. Whole-genome studies of paired samples from non-high-risk SMM patients that progressed to MM revealed a subset of patients in which the SMM clone was genomically indistinguishable from the clone at time of progression to MM [10]. A key determinant of progression in MM is therefore a permissive BM microenvironment. It is becoming increasingly apparent that immunotherapy may be the key to producing lasting remission and a functional cure in MM given that, just like MM, the immune system is “living” and has the ability to persist and adapt continuously and dynamically. Multiple strategies to reprogram the immune system against MM have been developed and many of them have unprecedented clinical efficacy even in the heavily pretreated relapsed refractory setting. However, the current state of immunotherapy in MM is still plagued by short-lived responses and a large reason is that MM creates an immunosuppressive BM tumor microenvironment, and studies have shown that the MM microenvironment is highly enriched for suppressive immune cells such as MDSCs, Tregs, pDCs, Bregs, N2 neutrophils, M2 macrophages, which leads to effector cell dysfunction and lack of persistence. A more in-depth understanding of the interactions between the MM and BM microenvironment is therefore necessary to identify and overcome immune escape mechanisms and develop novel therapies, as well as effective combinations of pre-existing therapies that will edge us closer to a cure for MM.

## Figures and Tables

**Table 1:** Summary of CAR T-Cell Clinical Trials in MM.

Product (Trial Name)	Target (Costim Domain)	Phase	Study Population	Response and Outcomes	Grade ≥3 CRS (Grade ≥3 NTX)	PMID (NCT)
**FDA approved**						
Ide-cel (KarMMa)	BCMA (4-1BB)	2; FDA approved	RRMM	ORR: 73%; mPFS: 12.1 months at 450 × 10^6^ target dose	6% (3%)	PMID: 33626253 (NCT03361748)
Cilta-cel (CARTITUDE-1)	BCMA (4-1BB)	1b/2; FDA approved	RRMM	ORR: 97%; mPFS and mOS not reached	5.1% (12.3%)	PMID: 35658469 (NCT04133636)
**BCMA CAR T-cell trials with reported data**						
CAR-BCMA first-in-human trial	BCMA (CD28)	1	RRMM	ORR: 81%; mPFS: 7.8 months	25% (4%)	PMID: 27412889 (NCT02215967)
CART-BCMA	BCMA (4-1BB)	1	RRMM	ORR: 48%; mPFS: 2.7 months	32% (12%)	PMID: 30896447 (NCT02546167)
Ide-cel + PI3K inhibitor (CRB-402)	BCMA (4-1BB)	1	RRMM	ORR: 55% (≥CR: 18%)	4.3% (6.5%)	(NCT03274219)
Cilta-cel (CARTITUDE-2)	BCMA (4-1BB)	2	RRMM and NDMM	ORR: 88.9% (≥ CR: 27.8%)	NA	(NCT4133636)
Zevor-cel (LUMMICAR-1)	BCMA (4-1BB)	1	RRMM	ORR: 87.5%; mDOR: 21.8 months	0% (0%)	(NCT03975907)
Zevor-cel (LUMMICAR-2)	BCMA (4-1BB)	1b/2	RRMM	ORR: 100%	0% (0%)	(NCT03915184)
Zevor-cel	BCMA (4-1BB)	1	RRMM	ORR: 87.5%; mPFS not reached	0% (4%)	(NCT03302403) (NCT03716856) (NCT03380039)
C-CAR088	BCMA (4-1BB)	1	RRMM	ORR: 95.2%; mPFS not reached	5% (0%)	(NCT04322292) (NCT03815383) (NCT03751293) (NCT04295018)
Single-domain antibody CAR	BCMA (4-1BB)	1	RRMM	ORR: 88.2%; mPFS 12.1 months;	2.9% (0%)	(NCT03661554)
FCARH143	BCMA (4-1BB)	1	RRMM	ORR: 100%; mPFS not reached	0% (0%)	(NCT03338972)
Orva-cel (EVOLVE)	BCMA (4-1BB)	1/2	RRMM	ORR: 91%; mPFS not reached	2% (4%)	(NCT03430011)
MCARH171	BCMA (4-1BB)	1	RRMM	ORR: 64%; mPFS not reached	20% (0%)	(NCT03070327)
FHVH33	BCMA (4-1BB)	1	RRMM	ORR: 80%	7%	(NCT03602612)
LCAR-B38M	BCMA (4-1BB)	1	RRMM	ORR: 88.2%; 12-month PFS: 52.9%	41.2% (0%)	(NCT03090660)
LCAR-B38M (LEGEND-2)	BCMA (4-1BB)	1/2	RRMM	ORR: 88%; mPFS: 15 months	7% (0%)	PMID: 30572922 (NCT03090659)
tEGFR suicide switch CAR	BCMA (4-1BB)	1	RRMM	ORR: 86%; mPFS not reached	0% (0%)	(NCT03093168)
KITE-585	BCMA (CD28)	1	RRMM	ORR: 100%; mPFS 1 month at 1 × 10^9^ dose	0% (0%)	PMID: 34249462 (NCT03318861)
Equecabtagene Autoleucel (FUMANBA-1)	BCMA	1/2	RRMM	ORR: 94.9% (≥CR: 68.4%)	0% (0%)	ChiCTR1800018137 (NCT05066646)
P-BCMA-101 (PRIME)	BCMA (4-1BB)	1/2	RRMM	ORR: 57%; mPFS not reached	2% (0%)	(NCT03288493)
CTL119	BCMA, CD19 (4-1BB)	1	High-risk MM	ORR: 80%	0%	(NCT03549442)
CART-19/BCMA (cocktail)	BCMA, CD19 (OX40/CD28)	1/2	High-risk NDMM	ORR: 100%; mPFS not reached	0% (0%)	PMID: 35114022 (NCT03455972)
SZ-MM-CART01	BCMA, CD19, CD138 (OX40/CD28)	1	RRMM	ORR: 100%	34%	(NCT03196414)
ARC-101	BCMA (4-1BB)	1	RRMM	ORR: 100%	4.8% (0%)	(NCT04155749)
ALLO-715	BCMA	1	RRMM	ORR: 60% in 320 × 10^6^ dose	0% (0%)	(NCT04093596)
**CAR T cells against other targets**						
CTL019	CD19 (4-1BB)	1	RRMM	ORR: 80%	0% (0%)	PMID: 29669947 (NCT02135406)
Tisa-cel	CD19	2	RRMM	NA	NA	(NCT02794246)
Various	SLAMF7	1/2	RRMM	NA	NA	(NCT03710421) (NCT04142619) (NCT04541368) (NCT03958656) (NCT04499339)
MCARH109	GPRC5D	1	RRMM	ORR: 71%; mPFS not reached	6% (6%)	PMID: 36170501 (NCT04555551)
Various	GPRC5D	1	RRMM	NA	NA	(NCT05219721) (NCT05016778)
CAR T-138	CD138 (4-1BB)	1/2	RRMM	ORR: 80%	0% (0%)	(NCT01886976)
ALTCAR.CD138	CD138	1	RRMM	NA	NA	(NCT03672318)
CAR2 Anti-CD38 A2 CAR T Cells	CD38	1	RRMM	NA	NA	(NCT03464916)
CM-CS1 T cell infusion	NKG2D (Dap10)	1	RRMM	ORR: 0%	0% (0%)	PMID: 30396908 (NCT02203825)
NKR-2 (CYAD-01) (THINK)	NKG2D	1/2	RRMM	ORR: 0%	NA	(NCT03018405)
CAR T-TnMUC1	Tn-MUC1	1	RRMM	NA	NA	(NCT04025216)
MLM-CAR44.1 T cells	CD44v6	1/2	RRMM	NA	NA	(NCT04097301)
OPC-415	MMG49	1/2	RRMM	NA	NA	(NCT04649073)
**Dual-Targeting CAR T cells**						
GC012F	BCMA and CD19	1	RRMM	ORR: 93.8% at 3 × 10^5^/kg dose	7.1%	(NCT04236011) (NCT04182581)
Various	BCMA and CD19	1	RRMM	NA	NA	(NCT04412889) (NCT04795882) (NCT04162353) (NCT03706547) (NCT04714827)
AUTO2	BCMA and TACI	1/2	RRMM	ORR: 43%	0% (0%)	(NCT03287804)
Various	BCMA and TACI	1	RRMM	NA	NA	(NCT05020444) (NCT04657861)
Various	BCMA and SLAMF7	1	RRMM	NA	NA	(NCT04662099) (NCT04156269)
NA	BCMA and CD38	1/2	RRMM	NA	NA	(NCT03767751)

**Table 2:** Summary of BiTE Clinical Trials in MM.

Product (Trial Name)	Target	Phase	Study Population	Response and Outcomes	Grade ≥3 CRS (Grade ≥3 NTX)	PMID (NCT)
**BCMA BiTE trials with reported data**						
Elranatamab (MagnetisMM-1)	BCMA	1	RRMM	ORR:75%; mPFS: not published	0% (0%)	(NCT03269136)
Teclistamab or talquetamab + daratumumab (TRIMM-2)	BCMA or GPRC5D	1	RRMM	ORR: 78%; mPFS: not published	0% (0%)	(NCT04108195)
Pacanalotamab	BCMA	1	RRMM	ORR 70; mPFS: 23.5	2% (4%)	PMID: 31895611 (NCT02514239)
Alnuctamab	BCMA	1	RRMM	ORR 83%; mPFS: not published	5% (0%)	NCT03486067
TNB-383B	BCMA	1	RRMM	ORR 52%; mPFS: not published	0% (0%)	NCT03933735
REGN5458 (LINKER-MM1)	BCMA	1/2	RRMM	ORR 60%; mPFS: not published	0% (0%)	NCT03761108
Pavurutamab + pomalidomide (ParadigMM-1B)	BCMA	1/2	RRMM	ORR 82%; mPFS: not published	7% (0%)	NCT03287908
Teclistamab (MajesTEC-1)	BCMA	2	RRMM	ORR 65%; mPFS: not published	0% (0%)	NCT04557098
**Non-BCMA BiTE trials**						
Talquetamab (MonumenTAL-1)	GPRC5D	1	RRMM	OR: 63%; mPFS not Published	4% (0%)	NCT03399799
Talquetamab + anticancer drugs (MonumenTAL-2)	GPRC5D	1	RRMM	NA	NA	NCT05050097
Talquetamab	GPRC5D	1	RRMM	NA	NA	NCT04773522
Talquetamab	GPRC5D	2	RRMM	NA	NA	NCT04634552
Blinatumomab	CD19	1	RRMM	NA	NA	NCT03173430
AMG 424	CD38	1	RRMM	NA	NA	NCT03445663
Y150	CD38	1	RRMM	NA	NA	NCT05011097
GBR1342	CD38	1	RRMM	NA	NA	NCT03309111
Cevostamab (CAMMA 1)	FcRH5	1	RRMM	NA	NA	NCT04910568
Cevostamab	FcRH5	1	RRMM	ORR: 52%; mPFS not reached	2% (0%)	NCT03275103

**Table 3:** Summary of Major Clinical Trials Leading to FDA Approval of Daratumumab.

Trial Name	Intervention	Study Population	Median Progression Free Survival (Months)	Overall Response Rate (Percent)	Median Overall Survival
Daratumumab for Treatment of Relapsed or Refractory Multiple Myeloma					
SIRIUS	Dara monotherapy	≥2–3 lines of therapy including PI and IMiD	3.7	29.2	1-year: 65%
CASTOR	Dara-Vd versus Vd	≥1 lines of therapy with response and progression	16.7 versus 7.1	83.0 versus 63.0	NR (at 3 years: 61% vs. 51%)
POLLUX	Dara-Rd versus Rd	≥1 lines of therapy with response and progression	44.5 versus 17.5	92.9 versus 76.4	NR (at 42 months: 65% vs. 57%)
APOLLO	Dara-Pd versus Pd	≥1 lines of therapy including lenalidomide and a proteasome inhibitor	12.4 versus 6.9	N/A (at 16.9 months 69 versus 46)	Overall survival data were not mature
CANDOR	Dara-Kd versus Kd	1–3 lines of prior therapy	NR versus 15.8	84.3 versus 74.7	NR
Daratumumab for Treatment of Newly Diagnosed Multiple Myeloma					
MAIA	Dara-Rd versus Rd	Newly diagnosed transplant-ineligible multiple myeloma	NR versus 33.8	92.9 versus 81.3	NR
ALCYONE	Dara-VMP versus VMP	Newly diagnosed transplant-ineligible multiple myeloma	36.9 versus 19.3	90.9 versus 73.9	NR
CASSIOPEIA	Dara-VTd versus VTd	Newly diagnosed transplant eligible multiple myeloma	NR vs. 46.7 months	sCR 29 vs 20	NR
GRIFFIN	Dara-VRd versus VRd	Newly diagnosed transplant eligible multiple myeloma	NR (at 24 months: 95.8 vs. 89.8)	sCR 42 vs 34	Overall survival data were not mature

**Table 4:** Summary of Major Clinical Trials Leading to FDA Approval of Isatuximab.

Trial Name	Intervention	Study Population	Median Progression Free Survival (Months)	Overall Response Rate (Percent)	Median Overall Survival
ICARIA-MM	IPd versus Pd	≥2 prior lines including IMid and PI	11.5 vs. 6.5	60.4 versus 35.3	At 12 months: 72% versus 63%
IKEMA	IKd versus Kd	1–3 lines of prior therapy, no prior carfilzomib	NR vs. 19.2	87 versus 83	Overall survival data were not mature

**Table 5:** Summary of Major Clinical Trials for FDA Approval of Elotuzumab.

Trial Name	Intervention	Study Population	Median Progression Free Survival (Months)	Overall Response Rate (Percent)	Median Overall Survival
ELOQUENT-2	ERd versus Rd	≥1 lines of therapy	19.4 vs. 14.9	79% vs. 66%	NR
ELOQUENT-3	EPd versus Pd	≥2 lines of therapy therapies, including lenalidomide and a proteasome inhibitor	10.25 vs. 4.67	53.3% vs. 26.3%	NR

**Table 6:** Summary of Major Clinical Trials for FDA Approval of Belantamab.

Trial Name	Intervention	Study Population	Median Progression Free Survival (Months)	Overall Response Rate (Percent)	Median Overall Survival
Dreamm-2	Belantamab	≥4 prior therapies, including an anti-CD38 monoclonal antibody, a proteasome inhibitor, and an immunomodulatory agent	5.7	31	NR
